# Selective Capsulotomies and Partial Capsulectomy in Implant-Based Breast Reconstruction Revision Surgery

**DOI:** 10.1155/2024/9097040

**Published:** 2024-02-27

**Authors:** Pietro Susini, Gianluca Marcaccini, Francesco Ruben Giardino, Mirco Pozzi, Francesco Volanti, Giuseppe Nisi, Roberto Cuomo, Luca Grimaldi

**Affiliations:** Plastic Surgery Unit, Department of Medicine, Surgery and Neuroscience, University of Siena, Policlinico le Scotte, Via Bracci 16, Siena 53100, Italy

## Abstract

**Background:**

Breast cancer with about 2.3 million diagnoses and 685,000 deaths globally is the most frequent malignancy in the female population. Continuous research has led to oncological and reconstructive advances in the management of breast cancer, thus improving outcomes and decreasing patient morbidity. Nowadays, the submuscular expander and prosthesis (E/P) implant-based breast reconstruction (IBR) accounts for 73% of all reconstructions. Despite its widely accepted efficacy, the technique is not free from complications and up to 28% of cases require revision surgery for mechanical complications such as capsular contracture, implant displacement/rotation, and implant rupture. With this study, the authors report their experience in the management of E/P IBR revision surgery through the technique of Selective Capsulotomies (SCs) and Partial Capsulectomy (PC).

**Methods:**

A retrospective study was conducted on patients who had previously undergone E/P IBR and presented for revision reconstruction between January 2013 and May 2023 at the Department of Plastic Surgery of the University of Siena, Italy. Reasons for revision included capsular contracture, implant displacement/rotation, and implant rupture. Revision reconstructions involved SC and PC with implant replacement. Fat grafting was also considered. The complication rate was evaluated by analysis of patients' medical records. Patients' satisfaction with the treatment was assessed through a specific questionnaire.

**Results:**

32 patients underwent revision surgeries. No early complication occurred. Recurrence rate was assessed at 19% with average follow-up of 59 months (range: 13–114 months). The average time between revision surgery and recurrence was 3 years (range: 1–6 years). 23 patients answered the questionnaire and were overall satisfied with the treatments (8.29/10).

**Conclusions:**

SC possibly associated to PC is a valuable option for E/P IBR revision surgery with minimal complications, reduced surgical trauma, short operating time, and relatively low recurrence risk. In addition, treated patients are overall satisfied with the results over time.

## 1. Introduction

Breast cancer is the most frequent malignancy in the female population with over 2.3 million new diagnoses in 2020 and 685,000 deaths globally [1, 2]. Following mastectomy, although numerous autologous and heterologous techniques have been described, implant-based breast reconstruction (IBR) represents the most widespread strategy [3–5], and skin or nipple-sparing mastectomies followed by immediate IBR represent 81.9% of all breast oncoplastic procedures [6].

Among IBRs, the expander and prosthesis (E/P) technique for submuscular IBR, first introduced by Radovan in the 1980s, continues to be the most popular and now accounts for 73% of all reconstructions [7, 8]. The literature agrees on the effectiveness of the technique; however, complications have been described in up to 30% of cases [9]. These include seroma, hematoma, infection, capsular contracture, implant malposition and displacement, breast asymmetry, improper contour, animation deformity, implant rupture, and prosthesis failure [9, 10]. Established risk factors include elevated BMI, tobacco use, poorly controlled diabetes mellitus, and radiotherapy [11, 12]. Such complications represent a major concern for both patients and surgeons, with up to 28% of cases requiring revision surgery over time [13, 14]. Worse data are reported for radio-treated patients, up to 51.5% of reoperation rate and 40% of reconstructive failure [15, 16]. Additionally, the presence of scar tissue, adhesions, and fibrosis from previous surgeries make revision reconstructions technically challenging.

Proposed techniques for revision surgery include percutaneous suture, internal capsulorrhaphy, fat grafting, partial or total capsulectomy, acellular dermal matrix- (ADM-) assisted reconstructions, and implant transposition in a prepectoral or partially prepectoral pocket with or without implant replacement [17–24]. However, there is little evidence of superior results for one technique over another [17–22]. Indeed, secondary reconstructions are still an open debate, and it is still a challenge to achieve aesthetically pleasing and long-lasting results. At time of implant replacement, invasive approaches that involve complete or large dissections of the periprosthetic capsule may lead to increased surgical trauma, intercostal muscle injury, pneumothorax, bleeding, and hematoma. These factors may act as an instigator for recurrence [25, 26]. Accordingly, if feasible, less invasive strategies should be considered.

With this study, the authors report their experience in the management of E/P IBR revision surgery through the technique of Selective Capsulotomies (SCs) and Partial Capsulectomy (PC) with implant replacement. SC and PC, when properly carried out, allow to model the prosthetic pocket in the desired way with minimal invasiveness, limited risks, and little lengthening of the surgical time. Study procedure, surgical technique, and outcomes are discussed.

## 2. Patients and Methods

A retrospective chart review was conducted of patients who underwent SC and/or PC revision surgery between January 2013 and May 2023 following E/P IBR. Any elective surgical procedure undertaken with the goal of altering, maintaining, or improving the previous E/P reconstruction outside of the expected course of recovery was termed revision surgery. Clinical evaluation and mutual agreement between patient and surgeon determined the need for a revision. Patient demographics, reasons for revision, and postrevision complication rate were obtained from reevaluation of patients' medical records.

Inclusion criteria consisted of patients with a history of IBR-related complications such as implant displacement/rotation, capsular contracture, and implant rupture that required revision surgery. Moreover, additional inclusion criteria were previous skin or nipple-sparing mastectomy followed by E/P reconstruction and at least 1 year of follow-up after revision surgery. Patients who had undergone autologous reconstruction, direct-to-implant (DTI) reconstructions, ADM-assisted IBR, and breast augmentation were excluded from the study. The characteristics of the implants replaced in revision surgery were compared to the previous ones in terms of implant volume, shape (round vs anatomical), and texturization (smooth vs textured).

Postoperative outcomes, complications, and recurrence rates were analyzed. Patients' satisfaction with the surgical procedure was also evaluated through a specific questionnaire designed for the study. The questionnaire was administered during a follow-up visit in May 2023. Any complaint following the revision reconstruction was also evaluated.

The aim of this article was to evaluate the use of SCs and/or PC for E/P IBR revision surgery. Conversely, the primary IBR strategy was not an end point of the study and was not further investigated or compared with alternative techniques.

The study followed the principle of the Declaration of Helsinki. All enrolled patients received detailed information on the retrospective chart review. All signed informed written consent for analysis and publication of personal data including photographic documentation.

### 2.1. Surgical Technique

**Figure 1:**
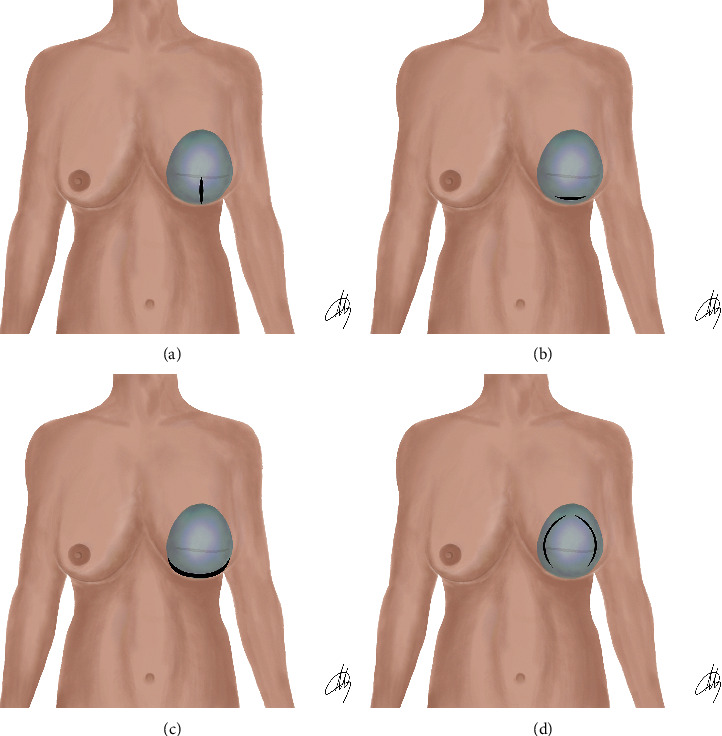
Schematic representation of selective capsulotomies. (a) Inferior vertical capsulotomy (IVC). (b) Inframammary fold capsulotomy (IFC). (c) Inferior semicircular capsulotomy (ISC). (d) Circumferential capsulotomy (CC).

Revision reconstructions involved SCs and/or PC with implant replacement, without implant plane change. The implant was accessed and removed through the previous mastectomy or E/P reconstruction scars. SCs were performed according to the technique described in a previous paper in 2010 [27]. The peculiarity of the technique is that of performing selective incisions of the periprosthetic capsule to remodel the definitive implant pocket in the desired way (Figure 1). SCs included the inframammary fold capsulotomy (IFC), which is realized at the level of the inner inferior reflection of the implant site and allows for lower placement of the inframammary fold but little gain in projection of the areolar area. The inferior semicircular capsulotomy (ISC), being sensibly longer than IFC, does not change the inframammary fold location but promotes a more evident convexity of the lower breast aspect and a more evident projection of the areolar area. The inferior vertical capsulotomy (IVC), by means of an incision along the midline of the breast, from 2 cm above the inferior internal fold to the limit of the areolar area, promotes better compliance and convexity of the lower lateral quarters. Finally, the circumferential capsulotomy (CC), through extensive incisions, allows the expansion of both upper and lower poles.

If there was a particularly thick anterior capsule, the SC was associated to a PC. In these cases, special care must be taken to minimize surgical damage to the pericapsular tissues. SC associated with PC allowed for even greater remodeling of the pocket, while maintaining minimal invasiveness. Implant plane change is excluded in order to minimize surgical invasiveness and guarantee adequate thickness between the prosthesis and the skin, except in particular cases, such as animation deformity.

In some cases, further improvement in breast symmetry has been achieved through adjunctive treatments including contralateral aligning mastopexy, inframammary fold repositioning, surgical scar revision, nipple-areola complex reshaping, and fat grafting. The latter is usually planned at a later time, at least 6 months after the operation, to improve the contour of the breast after the pocket has adapted to the new prosthesis.

All patients received antimicrobic prophylaxis of amoxicillin/clavulanate: 2.2 g intravenously and intraoperatively, and 1 g orally, twice a day, during the subsequent 5 days. No drainages were employed. Compressive elastic dressings were applied in all cases for 2 days and then substituted by anatomical bras.

### 2.2. Questionary

The following questionnaire was administered during a follow-up visit in May 2023 to all patients to assess their satisfaction and long-term response to the IBR revision reconstruction.

1. Are you generally satisfied with the treatment, 10 being best and 1 being worst?
2. How would you rate your final result aesthetically, 10 being best and 1 being worst?
3. How would you rate your result compared with your initial breast reconstruction, 10 being most improved, 5 being equal, and 1 being worse?
4. Are the required effects of breast revision reconstruction still maintained, 10 being identical and 1 being completely changed?
5. How would you rate your recovery, 10 being easiest and 1 being most difficult?

Patients were able to answer the questionnaire anonymously. Patients were also asked to report any critical issues on the treatment.

## 3. Results

### 3.1. Study Population

From January 2013 to May 2023, 32 patients underwent IBR revision surgery at the Department of Plastic Surgery of the University of Siena, Italy, for complications of previous E/P IBR. The revision surgeries were performed by multiple surgical teams in which the lead author participated. The characteristics of the patients are summarized in Table 1. The average age was 58 years old (range: 44–80), and the average BMI was 22.7 (range: 19.1–29.7). Of those participants, originally, 24 had bilateral reconstructions, 16 (50%) had contralateral mastopexy, and 8 (25%) had contralateral implants for symmetry. 23 (72%) originally had a skin-sparing mastectomy and 9 (28%) a nipple-sparing mastectomy. 6 (19%) received radiotherapy, 2 (6%) suffered from diabetes, and 14 (44%) were smokers. The average time between E/P IBR and revision reconstructions was 8 years (range: 1–28). The causes responsible for revision reconstructions are summarized in Chart 1. In particular, 9 (28%) patients complained of severe capsular contracture, 9 (28%) of implant displacement/rotation, and 3 (9%) of implant rupture. Additionally, 11 (35%) patients presented challenging mixed clinical pictures of severe contracture or implant rotation/displacement associated to implant rupture. No cases of animation deformity were detected in enrolled patients.

Different techniques have been used for the revision reconstructions (Chart 2). The procedure consisted of SC in 78% of cases. Of these, 48% were IFC, 28% ISC, 16% IVC, and 8% CC. SCs were associated to concomitant PC in 22% of cases. Autologous fat grafting was used in 15 cases (47%) to enhance breast volume and shape at revision surgery.

In each revision surgery, the prosthetic implants were replaced. The comparison between old and new implants is reported in Table 2. All implants were originally placed in a submuscular pocket. 89% implants were textured, and 11% were smooth. 88% implants were round, and 12% were anatomical. The mean primary implant volume was 343cc (range: 120−530cc). At time of revision surgery, 67% of final implants were larger in volume (final implant mean volume: 387cc, range: 240–540), 24% were smaller, and 9% were of equivalent size to the implants removed. Regarding the final implant surface, all implants were textured. The shape was also considered. 28 (88%) definitive implants were anatomical, and 4 (12%) were round. The submuscular prosthetic pocket was reshaped and contoured but not changed (submuscular vs subglandular) in any procedure. The main prostheses selected for revision surgery were Mentor CPG Low/Medium Height, Moderate Plus Profile, High Projection (47%), and Allergan 410 Textured Low/Moderate Heigh–Full/Extra Full Projection (34%).

**Figure 2:**
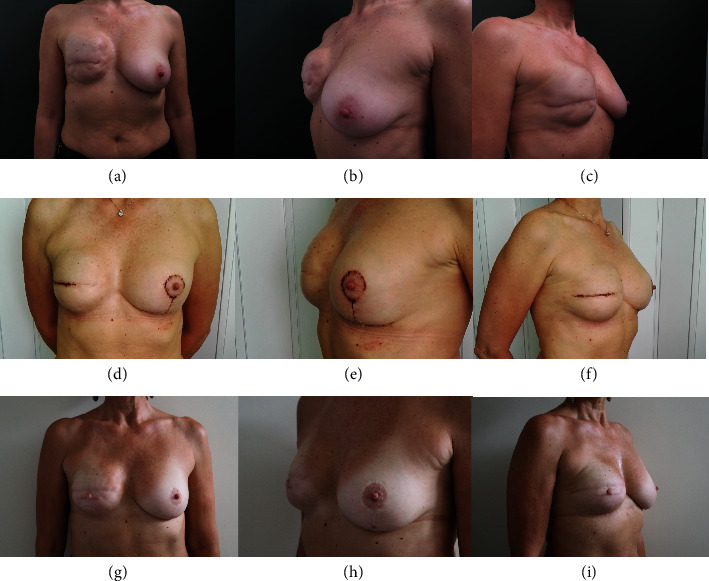
54-year-old woman who underwent ISC-SC revision surgery and contralateral mastopexy for severe capsular contracture two years after skin-sparing mastectomy and E/P reconstruction. (a–c) Preoperative images. (d–f) Follow-up 21 days after surgery. (g–i) Follow-up at 1 year.

**Figure 3:**
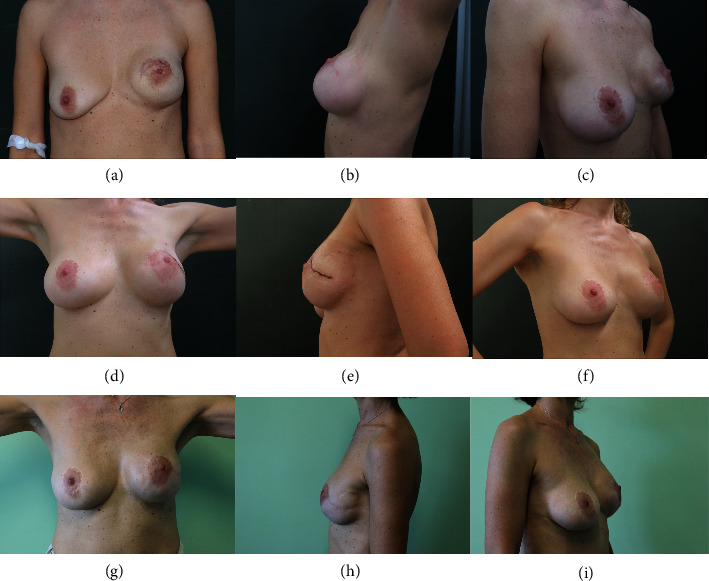
48-year-old woman who underwent IFC and medial-superomedial PC revision surgery for severe implant displacement two years after nipple-sparing mastectomy and E/P reconstruction with contralateral breast augmentation. (a–c) Preoperative images. (d–f) Follow-up 21 days after surgery. (g–i) Follow-up at 4 years.

8 patients (25%) were discharged the first postoperative day, 17 patients (53%) the second, and 4 (13%) the third. Only 3 patients (9%) were discharged on day 4. All patients received at least 1 year follow-up after surgery, with average follow-up of 59 months (range: 13–114 months) (Figures 2 and 3).

### 3.2. Outcomes

No early postoperative complications have been recorded. No wound dehiscence, delayed healing, skin necrosis, hematomas, seromas, or infections occurred. Two patients complained of moderate to severe pain after surgery, yet they recovered unremarkably. 6 patients (19%) had capsular contracture recurrence and required tertiary revision. Two of them had previously undergone radiotherapy; specifically, the rate of tertiary capsular contracture in radio-treated patients was 2/6 (33%) while in non-radio-treated patients, it was 4/26 (15%). The average time between revision surgery and recurrence was 3 years (range: 1–6 years).

In May 2023, the previously described questionnaire was distributed to all subjects of the study. 23 patients (72%) responded and were generally happy with their results (Chart 3).

## 4. Discussion

E/P IBR is the most common breast reconstruction technique, and numerous studies attest good functional and aesthetic results [28, 29]. Given the high number of procedures, inevitably, the prevention and treatment of E/P IBR complications is a topic of growing interest. Mechanical complications such as capsular contracture, implant displacement/rotation, and implant rupture are common after E/P reconstructions for numerous reasons. First, any implanted foreign body induces some degree of chronic tissue reaction and fibrosis with unpredictable outcomes [30]. Secondly, E/P IBR necessarily determines a conspicuous surgical trauma which is a trigger for complications [31]. Moreover, some patients are treated with radiation therapy, which further increases the risk [32, 33]. In particular, Couto-González et al. [15] showed a 51.5% of reoperation rate for patients who had undergone radiotherapy, while Nava et al. [16] reported up to 40% of reconstructive failure.

Our cohort included a 19% of radio-treated patients. However, this number may not reflect the burden of radio-treated patients who develop capsular contracture. Indeed, in our center, autologous reconstruction is often discussed for patients who had undergone radiotherapy and E/P IBR and present with complications requiring revision surgery such as severe capsular contracture or implant displacement. By evaluating the risks and benefits together with the patient, we suggest the optimal strategy depending on the specific case [34, 35]. Finally, malposition of the expander has been documented following breast expansion, with the upper pole typically excessive, hyper projected, and cranialized relative to the lower pole [36]. Although efforts are made to correct the asymmetry at the time of E/P replacement, there is still a risk of recurrence and implant displacement over time.

As a possible solution to these concerns, capsulotomy has been described in the management of capsular contracture in patients undergoing breast augmentation. It has also been described in E/P IBR to contour the expander pocket at the time of expander removal and definitive prosthesis placement [16, 27, 37, 38]. However, as far as we know, few data are available on its use in E/P IBR revision surgery. The main criticism of the technique is that the retained capsule could act as an instigator to recurrence [39]. By contrast, Hipps et al. [40], in a follow-up on 490 capsular contractures secondary to breast augmentation, showed comparable recurrence rates between capsulotomy (31%) and capsulectomy (34%). Additional studies showed that the capsule left in place is often partially/fully reabsorbed [39, 41]. Moreover, much more dissection is required for capsulectomy compared to capsulotomy [42], the replaced implant is covered by less remaining tissue, and there is a greater potential for muscle and nerve injury [42]. Fluid accumulation is also rare after a capsulotomy with observed rates of 1.3% for seroma [43]. Finally, twenty/thirty minutes operating time is usually sufficient for a unilateral capsulotomy compared to about 1 hour for a capsulectomy [42]. In light of these considerations, the question arises: is it really necessary to perform a capsulectomy?

There seems to be no harm in leaving thin, noncalcified capsules in place [44]. Our data showed clinical efficacy, few complications, and good results over time. The recurrence rate at 19% is in line with the data already present in the literature. The rate was higher in patients who had undergone radiotherapy; however, as far as we know, total capsulectomies do not correlate with better rates and autologous reconstruction is not always indicated or accepted. If the patient is a candidate for secondary IBR, we prefer to keep the surgery as minimally invasive as possible, performing SC or eventually a PC, but we avoid total capsulectomies unless the capsules are thick, calcified, or show infection signs.

Overall, SC and/or PC made it possible to avoid early complications while guaranteeing satisfactory results. Therefore, this conservative treatment should be preferred, while total capsulectomies should be reserved for the management of thick calcified or infected capsules in which capsule retention is not advisable [45–47].

## 5. Limitations of the Study

This study has several limitations starting from the limited sample number which prevents complete data reliability. It is agreed that larger scales would be a plausible next step. Although the purpose of the retrospective study was to describe the results of a technique, nonblind evaluation of patients has an inherent bias. The study does not control for the results of the traditional total capsulectomy in IBR revision surgery. Different types of prosthetic implants are used in terms of size, shape, texture, and brand. Follow-up was variable, and in some cases a longer period would have been appropriate to assess the effectiveness of the technique. The questionnaire is also a self-developed and nonvalidated test, therefore limited in its conclusions.

In addition, no patients were treated with ADM which is a bioactive scaffold capable of actively encourage cellular ingrowth, tissue regeneration, and angiogenesis [48–50]. ADM-assisted IBR is showing promising results with reported rate of 5% for postoperative complications and 2.1% for capsular contracture [21, 51, 52]. Specifically, a multicenter retrospective audit on Braxon ADM prepectoral breast reconstruction promoted by the Barcelona Hospital including data on 1450 procedures from 30 centers revealed a 2.1% of capsular contracture [51]. Furthermore, a recent systematic review and meta-analysis by Samuels et al. [53] in 2023 investigated the role of ADMs for the treatment of capsular contracture following breast augmentation procedures on a total of 481 breasts. The authors reported statistically significative reduced risk for capsular contracture recurrence following reconstructions with Strattice matrix. Specifically, the capsular contracture recurrence rate was 1.53% in the pooled data. It is noteworthy that the use of ADM is not precluded in our technique. It would be interesting to evaluate the effectiveness of ADM-assisted SC and PC IBR revision reconstructions through a true blinded, controlled trial. Future research in this area is expected.

Finally, no real statistical analysis has been performed to evaluate our outcomes with respect to the characteristics of the patients. However, due to the small cohort of enrolled patients, the data would still be unreliable. Nevertheless, this article demonstrates that in some cases, a minimally invasive, easy to perform, and safe technique leads to cost-effective results with great satisfaction for patients and surgeons.

## 6. Conclusions

The present study shows that SC possibly associated to PC is a valuable option for E/P IBR revision surgery with minimal risks and duration of surgery, no additional scars, few complications, and a relatively low recurrence risk. In addition, treated patients are overall satisfied and happy with the results over time. The strategy aims to minimize surgical invasiveness, postoperative pain, and reduce hospitalization times. It is relatively easy to perform and cost-effective surgery but potentially related to satisfactory results. Radio-treated patients may have a greater risk of recurrence, but that is similar to what occurs after more invasive procedures such as capsulectomy. The technique could also be combined with ADM application. These have been linked to promising results. We hope that future research will be conducted in this regard.

## Figures and Tables

**Table 1:** Patients' characteristics.

N. patients	32
Average age (range)	58 (44–80)
Average BMI (range)	22.7 (19.1–29.7)
Tobacco use	14 (44%)
Diabetes	2 (6%)
Radiotherapy	6 (19%)
N. unilateral reconstructions	8 (25%)
N. bilateral reconstructions	24 (75%)
N. skin-sparing mastectomies	23 (72%)
N. nipple-sparing mastectomies	9 (28%)

**Table 2:** Comparison between primary and final implants in the study population.

		Primary implants	Definitive implants	
Volume	Average	343cc	387cc	↑
	Range	120–530cc	340–540cc	

Shape	Round	5 (12%)	4 (11%)	↓
	Anatomic	27 (88%)	28 (89%)	↑

Texturization	Smooth	4 (11%)	0 (0%)	↓
	Textured	28 (89%)	32 (100%)	↑

Site	Submuscular	32 (100%)	32 (100%)	—
	Subglandular	0 (0%)	0 (0%)	—

## Data Availability

Data are available on request due to privacy/ethical restrictions.

## Supplementary Materials

Supplementary Materials Chart 1: causes responsible for IBR revision reconstruction in the study population. Chart 2: revision reconstruction technique in the study population. SC = selective capsulotomy; PC = partial capsulectomy; CC = circumferential capsulotomy; IVC = inferior vertical capsulotomy; ISC = inferior semicircular capsulotomy; IFC = inferior fold capsulotomy. Chart 3: responses of the study population to the questionnaire.
