# Supplementary material for: Selective Capsulotomies and Partial Capsulectomy in Implant-Based Breast Reconstruction Revision Surgery
Source: Breast J. 2024 Feb 27;2024:9097040. doi: 10.1155/2024/9097040 (PMC10914432; doi:10.1155/2024/9097040)
Supplement: Supplementary Materials — Chart 1: causes responsible for IBR revision reconstruction in the study population. Chart 2: revision reconstruction technique in the study population. SC = selective capsulotomy; PC = partial capsulectomy; CC = circumferential capsulotomy; IVC = inferior vertical capsulotomy; ISC = inferior semicircular capsulotomy; IFC = inferior fold capsulotomy. Chart 3: responses of the study population to the questionnaire. [file 9097040.f1.zip › Chart 1.docx]

**Chart 1.** Causes responsible for IBR revision reconstruction in the study population.
